# Hare-Lip in the Negro

**Published:** 1845-08

**Authors:** W. W. Comstock

**Affiliations:** Wrentham


### *Hare-lip in the Negro.*

To the Editor of the Boston Medical and Surgical Journal.

Sir,—I notice in your last number an extract from the Western Lancet, in which the inquiry is made, "Is the negro subject to hare-lip?" with a statement from the editor of that Journal, that, as far as his observation extends, although the deformity forces itself upon us very frequently, it is exclusively confined to the whites," and asks if there is a philosophical reason for the difference.

The question of philosophy can be answered by the fact that the difference does not exist. The African race is not exempt from that unfortunate deformity. I have myself witnessed more than one instance of its existence in the black, and recollect of having seen, a few years since, in the town of Paris, Oxford County, Me., a negro by the name of Hector Fuller, who had the misfortune of double hare-lip. I have the impression that instances of this kind are not very rare. Since writing the above, a member of my family informs me, that when passing from Boston to Portland, six or eight years ago, she met, on the deck of the steamer, a black man very badly deformed by double hare-lip, who, to use her own expression, was the most horrid-looking creature she had ever seen.

I am, Sir, your ob't serv't,

*Wrentham, July* 5, 1835. W. W. Comstock.

The Boston Journal of July 23d, contains a notice of two other cases of hare-lip in negroes; one a case of double hare-lip—so the question is solved.—Ed.

